# Supplementary figures and images for: Fipronil in sub-lethal doses leads to immuno-toxicological effects in broiler birds
Source: PLoS One. 2025 Jan 28;20(1):e0315915. doi: 10.1371/journal.pone.0315915 (PMC11774380; doi:10.1371/journal.pone.0315915)

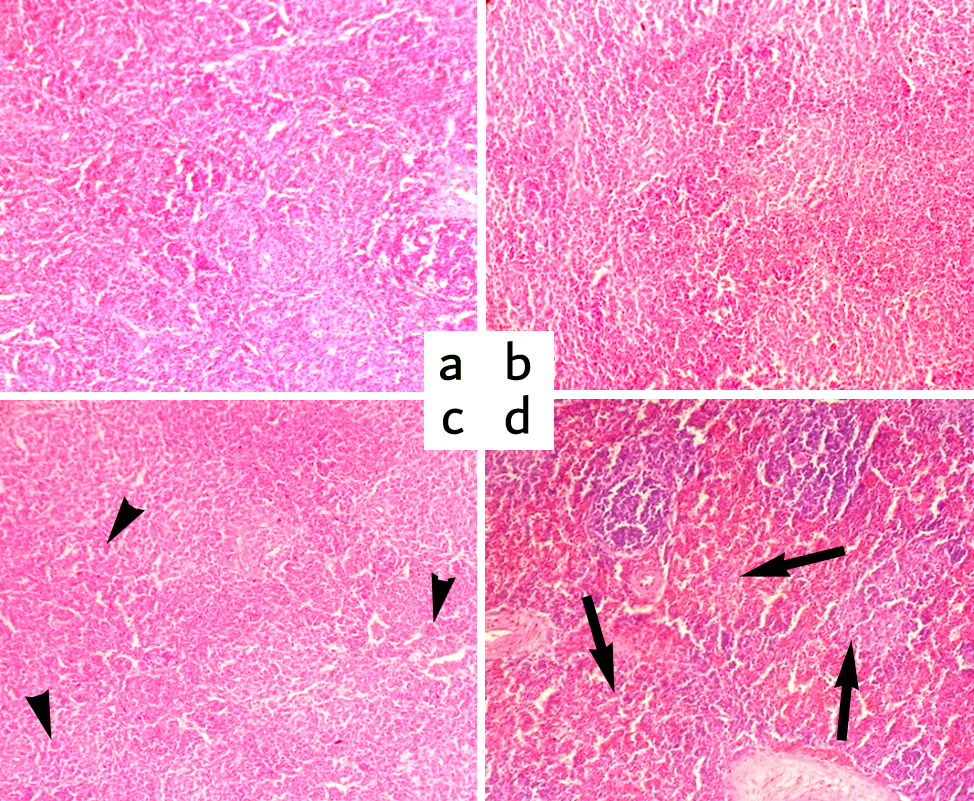

Supplement: S1 File — (ZIP) [file pone.0315915.s001.zip › 3-Fig-3.tif]

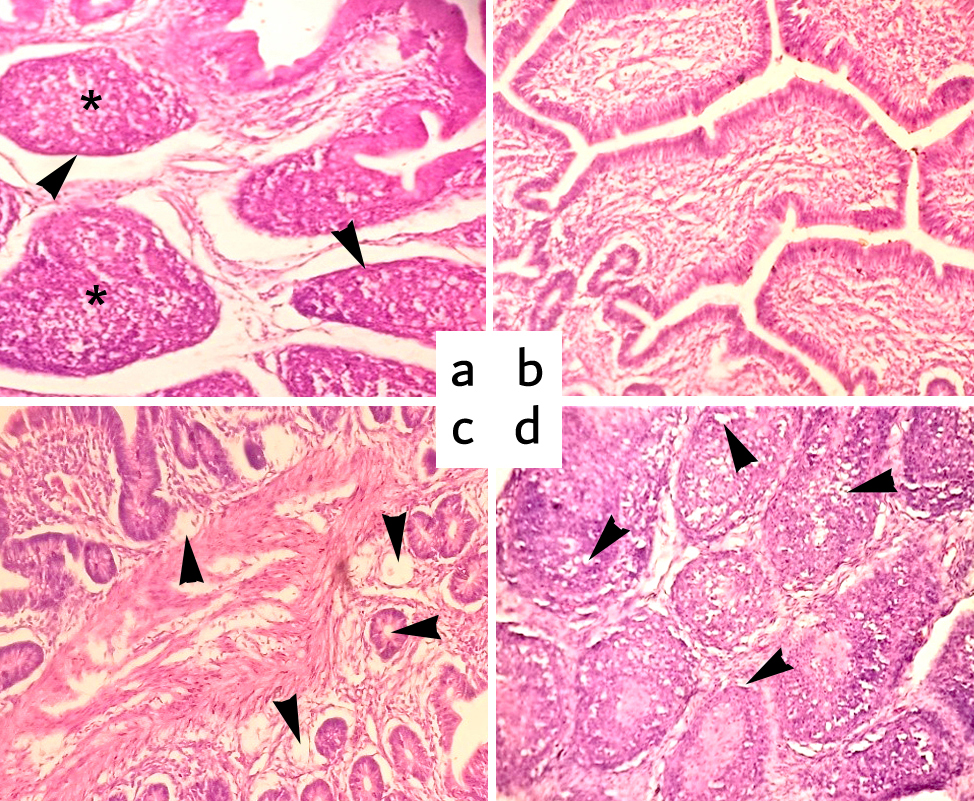

Supplement: S1 File — (ZIP) [file pone.0315915.s001.zip › 4-Fig-4.tif]
