# Supplementary material for: Fipronil in sub-lethal doses leads to immuno-toxicological effects in broiler birds
Source: PLoS One. 2025 Jan 28;20(1):e0315915. doi: 10.1371/journal.pone.0315915 (PMC11774380; doi:10.1371/journal.pone.0315915)
Supplement: Attachment — Submitted filename: PONE-D-24-46735_reviewer_1.pdf [file pone.0315915.s004.pdf]

FIPRONIL IN SUB-LETHAL DOSES LEADS TO IMMUNO-TOXICOLOGICAL EFFECTS IN BROILER BIRDS

Manuscript Number: PONE-D-24-46735

Dear Editor,

The data presented by the authors is relevant in the context of exploring the fipronil in sub-lethal doses leads to immuno-toxicological effects in broiler birds. The data is presented in a good scientific way and is understandable to common readers. The experimental design is excellent and covers all the necessary aspects. The references given in support of the data are advanced.

However, I have some trivial concerns regarding the manuscript. The points should be addressed in minor revision.

Line # 72-74. Repeated sentences.

Line # 103. Correct the word, "de-position"

Line # As the authors are going to evaluate sub-lethal dosage, therefore mention the LD_{50} for broilers with reference, to experimental birds and treatments sub-heading.

Line # 138-139. "Groups B, C, D, and E received fipronil @ 1.5, 2.5, 3.5, and 4.5 mg/kg body weight daily and these doses were selected on the basis of previous studies reported." Add the reference.

Line # 154-155. What is meant by post-treatment, please re-write the sentence and make it clear.

Line # 182 …… chicks/group received ink (1 mL/kg) injection…. Please mention the type of ink.

Line # 185-186. How much amount of blood samples were mixed in 4mL of 1% sodium citrate?

Line # 238. ……. response (4.00 ± 1.00, however, IgG in birds of Groups……….

Close the parentheses properly.

Line # 371-375. Re-write the sentence.

Line # 377 and many others. Write SRBCs instead of SRBC.

Line # 393. As only twice the word DTH has been used in the whole MS, therefore use its full form.

Line # 397-399. In these sentences, mention the treatment groups.

Line # 409. The result revealed…….. Correct as “the results reviled ………”.

Line # 412 and 415. Add suffix th with numbers.

Line # 438. Re-write the sentence.

Line # 444-446. Improve the sentence.

Line # Write the full name of *O. carvalhoi*
