# Supplementary material for: Fipronil in sub-lethal doses leads to immuno-toxicological effects in broiler birds
Source: PLoS One. 2025 Jan 28;20(1):e0315915. doi: 10.1371/journal.pone.0315915 (PMC11774380; doi:10.1371/journal.pone.0315915)
Supplement: Attachment — Submitted filename: PONE-D-24-46725_review.docx [file pone.0315915.s002.docx]

Manuscript: PONE-D-24-46725

Author: Shafia et al

General Comments: Interesting study with a lot of interesting data. My main concern is the appropriateness of using ANOVA with Tukey's post hoc test for a dose-response study. In dose-response experiments, where we’re evaluating graded responses to increasing doses of an additive or toxic compound like fipronil, contrasts such as linear or quadratic (or higher-order contrasts, if relevant) often provide a more meaningful analysis than pdiff. We can quantify the strength and nature of the dose-response relationship in a more biologically relevant manner. ANOVA with Tukey’s post hoc test is more appropriate for comparing unrelated groups rather than ordered dose levels. Furthermore, the authors should clearly state the trial novelty or research gap to be filled with this study, many of the citations references previous trials with overlapping objectives with the current study.

Specific Comments:

LN 24: I think you mean plant agriculture here, as opposed to animal agriculture.

LN 30: “maintained” at 37C

LN 72 – 74: Repetition

LN 126: Add brand identifiers/vendor for the fipronil used in this trial.

LN 130: Add chicken breed

LN 139: What type of mineral oil

LN 143: Curious there is no mortality recorded over a 45 day period for birds challenged multiple times.

Ln 154: How was blood collected? Please state the number of birds per replicate and how many were selected for sampling per phase.

Results and discussion: Rather than A,B—E, use better treatment descriptors here and in tables. It is easier for the readers to follow your study this way. Where necessary, combine common data into a single paragraph e.g. absolute/relative weights of organs can be combined into one or two succinct paragraphs.

Data table: Add exact p values for each response critetia, not a blanket p value in the footnote.
