# Supplementary material for: Fipronil in sub-lethal doses leads to immuno-toxicological effects in broiler birds
Source: PLoS One. 2025 Jan 28;20(1):e0315915. doi: 10.1371/journal.pone.0315915 (PMC11774380; doi:10.1371/journal.pone.0315915)
Supplement: Attachment — Submitted filename: Fipronil-Plos-One-08102024-Gul.docx [file pone.0315915.s003.docx]

**FIPRONIL IN SUB-LETHAL DOSES LEADS TO IMMUNO-TOXICOLOGICAL**

**EFFECTS IN BROILER BIRDS**

**Abstract**

Pesticides, including fipronil, are used mainly in agriculture; however, in veterinary and animal husbandry, their potential use is to control the pests responsible for vector-borne diseases. Their residues in agriculture products and direct use on farms are responsible for potentially harming livestock and poultry. So, this study was designed to evaluate the toxico-pathological effects of fipronil on the immune system of poultry birds. One hundred a-day-old chicks were purchased from a local hatchery, and standard housing conditions were provided from brooding till the end of the trail. The temperature was 37^ᴼ^C, and the humidity was maintained at 60-70%. Clean water and a basal diet were provided *ad libitum*. After three days of acclimatization, birds were divided into five experimental groups (A to E), each containing 20 birds. Group A was kept as a control group. Fipronil was administered orally through crop tubing @ 1.5, 2.5, 3.5, and 4.5 mg/kg to groups B-E, respectively. Birds were euthanized humanely on the 15^th^, 30^th^, and 45^th^ days of the experiment. Immunological parameters were evaluated, i.e., antibody titers against NDV and SRBC, phagocytic activity to clear carbon particles, avian incompetence to avian tuberculin, and histopathological alterations in the lymphoid organs.  The SAS^®^ University Edition software was used for data analysis. The results indicated decreased antibody titers against NDV in the treatment groups compared to the control. Similarly, antibody response to SRBC, phagocytic activity in clearing the carbon particles, and sensitivity to avian tuberculin in the treatment groups were also decreased. Results also revealed that the bursa of Fabricius thymus and spleen were also affected due to the toxic effect of fipronil, even at sub-lethal doses.

**Keywords:** Fipronil, Immunotoxicology, broilers, poultry, histopathology

**Introduction**

Pesticides are used in advanced health practices, agriculture, and animal husbandry. Around the globe, the extensive use of these chemicals poses a serious threat to the environment and human and animal health [1, 2]. Although due to such potentially harmful effects, pesticides and insecticides have been limited in many countries like the European Union, USA, and China gradually, but still in developing regions, they are being used extensively [3]. Fipronil is from the class of phenyl pyrazoles and was initially discovered in 1987 and was launched in 1993. The half-life of fipronil in granular form in aqueous form and soil is 125 and 438 hours, respectively. Being a soil or water contaminant, just like many other pesticides and insecticides, it has been reported to lead to oxidative stress and injuries in poultry, livestock, and humans [4, 5].

Fipronil is a wide-spectrum insecticide that hits aminobutyric acid (GABA)-gated chloride channels and causes insect death. The vertebrates' organs affected by fipronil subjection include the thyroid, spleen, kidney, and liver [6, 7]. The toxicity of fipronil is highly selective, and it has a better affinity than mammalian receptors with arthropod GABA. The links to the GABA receptor interfere with chloride channels, leading to loss of neuronal signals, over-awakening, and death [8]. There are three major pathways by which fipronil is metabolized in the environment through photolysis on the upper surface of the soil, hydrolysis, or oxidation, and leads to the formation of fipronil sulfide, fipronil amide, and fipronil sulfone, respectively. Except for fipronil amide, the other two are more persistent and toxic in the environment [9-11].

Due to its extensive application of insecticides/pesticides, such as pyrethroids, organophosphates, and carbamates in agricultural systems to control pests lead residues in crops, soil, groundwater, rivers, etc. This residual effect plays a pivotal role in environmental pollution and its potentially harmful effects on non-target species, including bees, vertebrates, aquatic species, and poultry. Many non-target species are part of food chains that severely threaten human health [3, 12]. Many non-target species are part of food chains that severely threaten human health [13]. Although fipronil is present in the environment at low concentrations, its chronic exposure is much more likely to occur than acute poisoning to non-target species that require a higher level to have an observable effect [5, 6, 14].

Many environmental samples contain the residues of fipronil or its metabolites, and they have been detected in soil, water, vegetables, and animal products due to the mixing of irrigation water with other sources of water [4, 10]. In animals, fipronil has been detected in birds' adipose tissue, brain, and liver after chronic exposure. It has been reported that birds exposed to a sublethal dose of fipronil result in a histopathological alteration in the spleen, liver, and kidney and a genotoxic effect. Fipronil-associated damage to DNA and the histopathological changes in tissues, mainly at high doses, have been published [15, 16]. In a few experimental studies where fipronil toxicities were induced, higher morbidity and mortality rates have been recorded, along with reduced body size, decreased hatching, etc. In addition, heart deformities such as edema and irregular heartbeat have been documented due to abnormal blood flow [5, 17, 18].

In fish, fipronil exposure generally leads to erythrocyte injury, immune system suppression, tissue physiology disruption, and death [19]. Fipronil has been declared harmful to many birds and most fish species. This insecticide has sub-lethal effects that result in concentrations far below those associated with deaths, varying from genotoxic and cytotoxic impact, compromising the immune system and reproduction rate. Using fipronil as a seed treatment on some crops poses risks for small birds, and the intake of only a few treated seeds could lead to mortality or fertility problems for sensitive bird species [20].

Unwanted effects on vertebrates, if exposed to fipronil, have been recorded like intense vascular congestion in the liver during histopathological evaluation, disrupts the immune system, leading to decreased cellular immunity and reduction in the weight of both lymphoid organs, spleen, and thymus concerning organ to body weight ratio [21-23]. It has been reported that if mothers are exposed to fipronil, it leads to the marked induction of apoptosis in the offspring's thyroid and thymus [24, 25]*.* In mammals, it has been reported that fipronil results in a decline in immunoglobulin M (IgM) levels and different histopathological alterations [26].

Histopathological alteration has also been observed in the vertebrates' liver, gill, kidney, and brain. The liver displayed other damages, including de-position pyknosis, steatosis, hypertrophy, sinusoidal dilation, and glycogen [3, 27]. The most critical damages found were renal tubular degeneration, sinusoidal dilation, systemic changes, and hemolysis in kidneys. These effects were more apparent in the maximal dose, while the duration of exposure to this organ had less impact on fipronil toxicity [28].

Even though it is used extensively in agriculture, fipronil is also used in veterinary medicine as a broad-spectrum insecticide to kill ectoparasites, including lice, beetles, ticks, and cockroaches. However, it led to a global food safety incident in 2017. It was found in chicken eggs in more than 40 countries [12, 29]. Residues in milk and meat of bovines have also been detected. Considering all these harmful effects at toxic levels, the current study was planned to evaluate the potential harmful effects at sub-lethal dosage levels, being a food chain contaminant in broiler birds that are being used as a good and cost-effective source of animal protein in Pakistan.

**Materials and methods**

**Ethical Approval**

This experimental study was conducted after the through approval from the Graduate Studies and Research Board, University of Agriculture, Faisalabad (UAF), Pakistan vide letter No. 10917-20 Dated 14-05-2020. All the husbandry practices and euthanasia was performed keeping in view all considerations of animal welfare.

**Chemical**

Fipronil (C_12_H_4_Cl_2_F_6_N**)** belongs to the phenylpyrazole chemical family, and it was purchased from a local market in Faisalabad, Pakistan, with a purity of 30 % and stored at room temperature.

**Experimental birds and treatments**

One-day-old broiler chicks (100) were purchased from the hatchery and maintained in a standard housing environment. The temperature was maintained at 37℃ and gradually decreased to 25℃ till the end of the experiment with 60-70% humidity. At the start of experiment 22h of light was provided and later on 20h till the end of the experiment. Clean water and a basal diet were provided (*ad libitum*).

Birds were vaccinated for the diseases according to the prescribed schedule for broilers. All the birds were acclimatized for three days and then randomly divided into five experimental groups (20 birds/group). Group A served as control. Groups B, C, D, and E received fipronil @ 1.5, 2.5, 3.5, and 4.5 mg/kg body weight daily and these doses were selected on the basis of previous studies reported. The fipronil was dissolved in mineral oil and administered to each bird through a crop tube. Birds were monitored twice daily to observe behavioral changes, if any. To evaluate the humoral immune response, these birds were vaccinated against ND at 5, 23, and 28 days of age (ND Lasota strain, VRI, Lahore, Pakistan). This trial duration was 45 days. During this trial, no mortality was recorded. Birds were shifted to necropsy unit in the Department of Pathology to collect the samples for the histopathology on 15, 30 and 45^th^ days of experiment. Euthanasia was performed as per the recommendation of the animal ethics committee of the University of Agriculture, Faisalabad and later on necropsy was conducted as per the standard protocols for the poultry birds.

**Parameters Studied**

**Immunological studies**

**Antibody Titers against Newcastle Disease (ND) Virus**

The procedure already described was followed to determine the antibody titers against the ND virus [30]. Chicks were vaccinated against ND at the age of 2, 23, and 28 days to assess the humoral responses of the treated birds. Serum was extracted from blood samples collected on Days 14 and 21 and on and post-treatment Days 28 and 35. Serum samples were then subjected to hemagglutination inhibition (HI) assay [31] to determine the degree of antibody formation against the ND in these fipronil birds.

**Antibody titer against sheep RBCs**

The trial evaluated humoral immune responses by estimating antibody titer SRBCs [32, 33]. Briefly, 1mL of a 3% SRBCs suspension was injected I/V into three chicks from each treated group (i.e., primary dosing) at the age of Day 7. A booster dose was injected 14 days later in these birds (i.e., at Day 21 of age). The wing vein was used to collect blood without anti-coagulant on 7, 14, 21, and 28 days post-primary doses for serum collection. Each serum sample was stored at -20°C until analysis. Serum samples were also inactivated for 30 minutes at 56°C and then analyzed for total anti-SRBC antibodies following the method already described [32].

**Lymphoproliferative response to avian tuberculin**

The cell-mediated immune response was assessed through delayed-type hypersensitivity (DTH) response to avian tuberculin [34] through lymphoproliferative response. For this test, at day 40 of age, three birds/group received an injection (0.2mL avian tuberculin) into the intra-digital space between the 3rd and 4th digit of their right foot. Normal saline was injected into the same space in the left foot as the "self"-control. Skin thickness between the digits was measured 24, 48, and 72 hours post-injection. Thickness was also measured before either injection to provide a baseline value. The cutaneous hypersensitivity response at each time point was calculated.

**Carbon clearance assay**

A non-specific immune response was accessed through a carbon clearance assay for phagocytic activity to clear the carbon particles [35]. For this test, on day 14 of age, three chicks/group received ink (1 mL/kg) injection into the right-wing vein. An identical treatment was carried out on another subset set of three chicks/group at day 28 of age. On each day, blood samples were collected from the left-wing vein at 0 minutes, 3 minutes, and 15 minutes post-ink injection. Each blood sample was instantly mixed with 4mL of 1% sodium citrate and centrifuged for 4 minutes. The relative amount of carbon particles remaining in the supernatant was then measured with a spectrophotometer (Spectro 20D Plus RS-232C) at 640nm [35].

**Gross and Histopathology**

To evaluate toxicological effects on immune organs, including the Bursa of Fabricius, Spleen, and Thymus, these organs' absolute and relative weights were recorded on the 15, 30, and 45^th^ days of the experiment. For this purpose, birds from each group were euthanized humanely through cervical dislocation, and lymphoid organs were collected. Absolute and relative organ weights were calculated. Tissue samples collected from the above-mentioned lymphoid organs were collected and preserved for histopathology in a neutral 10% buffered formalin solution, dehydrated, embedded, mounted on glass slides, and stained with hematoxylin and eosin (H&E) staining method as per following the procedure already described [36, 37].

**Statistical Analysis**

Data thus collected during this experimental trial were subjected to ANOVA (analysis of variance) to determine the statistical difference between the means, and these means were compared using the Tukey post hoc test to determine the highest significant difference using SAS^®^ University Edition online software SAS stat 15.1. All the results obtained during the trial have been expressed as means with their standard error. Regarding the homogeneity of ANOVA, Levene's test indicated a non-significant change at a significance level of 0.05, which is considered homogeneous [38].

**Results**

**Immunological parameters**

**Antibody titers against Newcastle Disease Virus**

The results recorded on the 14^th^ and 21^st^ day of the experiment indicated that birds of Group A (control) had significantly (*p* < 0.05) higher antibody titers against NDV antibody titers against NDV as compared to birds in Groups C, D, and E. In contrast, these groups were not significant among each other. Birds of Group B had non-significant (*p* > 0.05) lower antibody titers against NDV as compared to control Group A (Table 1). Similar trends and results were observed on days 28^th^ and 35^th^, as shown in the data presented in Table 1.

**Total antibody titers against SRBCs**

After assessing the total antibody titers against SRBCs, the results indicated that on the 7^th^ day of primary injection, birds in control Group A showed the highest antibody titers against SRBCs. Birds of Group E had a significant (*p* < 0.05) decrease in antibody titers against SRBCs compared to birds in the control group. Groups B, C, and D Birds showed a non-significant decrease in antibody titers against SRBCs from the control group (Table 2). However, on the 7^th^ day of booster injection, the control group (A) led the highest antibody titers against SRBCs. At the same time, birds of Groups B, C, D, and E showed a significant (*p* < 0.05) decrease in antibody titers against SRBCs compared to birds in the control group. However, these birds in groups (B-E) had non-significantly different antibody titers against SRBCs to each other (Table 2).

On the 14^th^ day of primary and booster injection in birds of the control group (A) led to significantly higher antibody titers against SRBCs as compared to birds in Groups C, D, and E; however, these had non-significantly different antibody titers against SRBCs among each other. Group B had significantly (*p* > 0.05) lower antibody titers against SRBCs from the control group (Table 2).

**IgG response**

After the 7th day of primary injection, birds of the control group (A) showed the highest IgG response (4.00 ± 1.00, however, IgG in birds of Groups B, C, D, and E showed a non-significant difference as compared to the control group (Table 2). After the 14^th^ day of primary injection, birds in the control group showed the highest IgG response against SRBCs. Birds in Groups B, C, and D showed a non-significant decrease in IgG response as compared to control Group A, while Group E (1.33 ± 0.58) showed a significant (*p* < 0.05) reduction in IgG response as compared to birds in control and other groups (Table 2).

After the 7th day of booster injection, birds of the control group (4.33 ± 1.51) showed a higher IgG response; however, IgG in birds of Groups B, C, D, and E showed a non-significant difference as compared to the control group (Table 2). On the 14th day of booster injection, the highest IgG response was observed in birds of the control group, whereas Groups D and E had a significant (*p* < 0.05) decrease in IgG response compared to the control group. However, Groups B and C birds were non-significantly different from the control group (Table 2).

**IgM response**

As mentioned above, the IgM response was similar to that of IgG in fipronil-treated groups. Briefly, on day 7^th^, birds in Group E (1.66 ± 0.58) showed significantly (*p* < 0.05) the lowest IgM response compared to control birds in Group A (3.66 ± 0.57). Birds in Groups B, C, and D showed a non-significant decrease in IgM response compared to control group A. A similar trend was observed after the 14^th^ day of primary injection data (Table 2).

After day 7^th^ of booster injection, birds in Groups B, C, D, and E showed a non-significant (*p* > 0.05) decrease in IgM response against SRBCs compared to the control group. However, on the day 14^th^ of booster injection, birds in Group E (0.66 ± 0.58) showed significantly (*p* < 0.05) lower IgM response as compared to birds of control Group A. Birds in Groups B, C, and D showed a non-significant decrease in IgM response as compared to birds in the control group (Table 2).

**Incompetence to avian tuberculin**

Avian tuberculin was injected into the birds, and skin thickness (mm) was measured. The result indicated that after 24 hours, birds of Groups C, D, and E had significantly (*p* < 0.05) lower values as compared to birds in the control group (1.25 ± 0.12). Still, the response to avian tuberculin was non-significantly (*p* > 0.05) different from birds of fipronil-treated birds. The skin thickness of birds in Group B (1.04 ± 0.11) showed a non-significant decrease compared to birds of the control group. However, it has significantly (*p* < 0.05) higher skin thickness than groups C, D, and E (Fig. 1).

After 48 hours of avian tuberculin injection, birds of groups D and E had significantly (*p* < 0.05) lower thickness as compared to the birds in the control group (0.66 ± 0.16). Groups B and C birds showed a non-significant decrease in skin thickness compared to the control group. After 72 hours, significantly (*p* < 0.05), the lowest skin thickness values were recorded in group E (0.19 ± 0.04) as compared to all treatment groups. The birds in fipronil-treated groups had significantly (*p* < 0.05) lower skin thickness as compared to birds in the control group but were non-significantly different from each other (Fig. 1).

**Phagocytic activity through carbon clearance assay (CCA)**

Indian ink was injected in the wing vein of the birds to determine the phagocytic activity in clearing the carbon particles. Results obtained after 3 minutes indicated that birds in Group E (84.61 ± 1.82) had significantly (*p* < 0.05) higher values as compared to the birds in control Group A (46.16 ± 5.63) and other fipronil-treated groups (Fig. 2). Follow-up reading was recorded after 15 minutes. That indicated the highest carbon particles in Group E (81.57 ± 4.15) followed by Groups D and C; however, statistically, these values were non-significant to each other. However, birds in Group A (36.85 ± 2.52) showed significantly (*p* < 0.05) different Indian ink responses than all the fipronil-treated Groups, but a non-significant difference was recorded to that of Group B (Fig. 2).

**Immune organ weight**

**The absolute and relative weight of the spleen**

The results revealed that the absolute weight of the spleen was inversely proportional to the dose of the pesticide; as the dose of the pesticide increased, the weight of the spleen decreased. On day 15^th^ (0.53 ± 0.07), 30^th^ (1.88 ± 0.05), and 45^th^ (4.08 ± 0.06) weight of the spleen of control Group A had significantly (*p* < 0.05) higher weights as compared to other fipronil treated groups (Table 3). On the 15^th^, 30^th^, and 45^th^ day of the experiment indicated that the control group (group A) had non-significantly (*p* > 0.05) higher relative weight from all fipronil-treated groups, i.e., Groups B, C, D, and E (Table 3).

**The absolute and relative weight of the bursa of Fabricius**

The absolute weight of the bursa of Fabricius is also inversely proportional to the dose of fipronil, just like that of the spleen. On days 15^th^, 30^th^, and 45^th^, the bursa of Fabricius of control Group A had significantly (*p* < 0.05) higher weights as compared to other fipronil-treated groups (Table 3). The relative organ weight results recorded on day 15^th^ indicated that control Group A (0.18 ± 0.02) had significantly higher relative weight than Groups D and E and non-significantly higher than Groups B and C. On day 30^th^, control Group A had significantly (*p* < 0.05) higher relative weight than Group E, while significantly higher than Groups B, C, and D (Table 3). On day 45^th^, control Group A had significantly higher relative weight than Group E, while non-significantly different from Groups B, C, and D (Table 3).

**The Absolute and relative weight of the thymus**

The results obtained for the thymus were similar to those mentioned above in two lymphoid organs. Briefly, on days 15^th^, 30^th^, and 45^th^, absolute weights of the thymus of control Group A were significantly (*p* < 0.05) higher weights as compared to others (Table 3). The results on the relative weight of the thymus revealed that on days 15^th^ and 30^th^, control Group A had non-significantly higher relative weight than Groups B, C, D, and E. On day 45^th^, control Group A had significantly (*p* < 0.05) higher relative weight than Groups C, D, and E. In comparison, Group B had a non-significantly lower relative weight as compared to control Group A (Table 3).

**Histopathology of immune organs**

**Spleen**

Group A (Control) showed standard structure; no change was observed on the 15^th^, 30^th,^ and 45^th^ days. White pulp, red pulp, central arterioles, lymphoid follicle, and capsule showed no changes. Group B showed little disruption in central arterioles. However, the histological structure remains almost similar to the control group (A). Still, severe congestion was observed in Groups C and D. Group E, treated with a high dose of fipronil (4.5 mg/kg BW), showed lymphocyte depletion in the lymphoid follicles. The severity of the lesion increased in a dose-dependent manner and in the number of exposure days. Marked changes were recorded in the spleen of group E on the 30^th^ and 45^th^ day of the experiment (Fig. 3).

**Bursa of Fabricius**

Group A (Control) showed normal structure; no change was observed on experimental days. The lymphoid follicle showed the normal cortex, medulla, and cortico-medullary border. Group B showed mild congestion and disruption of the cortex, medulla, and cortico-medullary border. However, severe congestion and disruption were observed in groups C and D. In the high dose fipronil (4.5 mg/kg BW) treated (group E), moderate infiltration of inflammatory cells was observed on day 15^th^, laterally, vacuolar degeneration was also evident in the tissue collected on 30 and 45^th^ day of the experiment (Fig. 4). The severity of the lesions increased in a dose-dependent manner and duration of exposure. Marked histopathological changes were recorded in the bursa of the group E on the 30^th^ and 45^th^ day of the experiment (Fig. 4).

**Thymus**

Group A (control) showed a normal cell structure. No change was observed on days 15^th^, 30^th,^ and 45^th^. Clearly, demarcated Hassle's capsules were observed. Group B showed mild changes and disruption, almost similar to A group (control), but groups C and D showed severe congestion. Group E showed degenerative changes in the medullary region. In high-dose group E (fipronil: 4.5 mg/kg BW), marked histopathological changes in the thymus were recorded. On the 15th day, mild depletion of lymphocytic parenchyma was recorded, and on the 30^th^ day, it was accompanied by vacuolar degeneration. Inflammatory and histopathological changes were also evident on the 45^th^ day due to chronic exposure to fipronil.

**Discussion**

Poultry is a significant and vibrant agricultural sector in Pakistan. It contributes significantly to the national GDP. The poultry sector gained commercial significance in Pakistan in the 1960s; since then, it has gained a significant state. This is a major and cheapest source of protein and contributes to approximately 30 percent of the country's overall meat production [39-41]. The main grains used in poultry feed are maize, wheat, rice, etc. Being a vibrant industry, the agriculture sector is continuously increasing the production of these crops. To ensure better crop yield, agrochemical companies are producing new forms of chemicals, but the danger to the consumer must be noticed. Such chemicals leave their crop residues behind and eventually enter the end-user [42, 43]. Despite high environmental effects, fipronil is still used in agriculture and veterinary setups [10, 44]. Due to its mechanism of action through receptor targeting, particularly for γ-aminobutyric acid, it leads to neurotoxicity, cytotoxicity, nephrotoxicity, and reproductive toxicity in vertebrates [38, 45, 46].

In the current study, the effect of sub-lethal doses of fipronil was observed on the immunity and immune organs of the broiler birds. Significant results recorded during the trial have been discussed here. The antibody titers against NDV in fipronil-treated groups were significantly lower than those of the control group on the 14th, 21st, 28th, and 35^th^ days of the trial. The lowest titers were recorded in group E, followed by the others. These results show that at these sub-lethal dosages, the immune system of the birds has been depressed, which has been by previous studies that the fipronil has deleterious effects on the immune system of the vertebrates through depressed cellular immunity and results in immunosuppression and immunotoxicity [1, 19, 22].

Previously, humoral antibody production has been evaluated in different species through intravenous administration of SRBC in chicken [31-33] against different pesticides/insecticides and profound effects have been reported in a dose-dependent manner. In the current study, the effects of fipronil exposure on antibody titer against the SRBC were also evident in a dose-dependent manner. The lowest antibody titers were observed in high-dose treated group E on day 7^th^ and so on. These results were consistent with earlier reports [47, 48]. The antibody titer to SRBC was decreased due to inhibition of antigen by the reticuloendothelial system [49, 50] and due to inhibition of immunoglobulin synthesis and reduction of T-lymphocyte [48].

Skin thickness was the highest lymphoproliferative response in the A group at 24, 48, and 72 hours. At 24 and 48 hours and significantly higher than Group E. Lymphoproliferative response against avian tuberculin was decreased because the T-cell response was lower, as previously reported [31,47] against different xenobiotics including chlorpyriphos, arsenic and thiamethoxam (TMX), respectively. Intra-dermal injection of avian tuberculin stimulates the T and B cells. It increases the production of cytokines /chemokines and the recruitment of immune cells (macrophages, neutrophils, etc.), so skin thickness increases at the injection site [51].

In terms of DTH immune response, it was observed that birds from control Group A had increased phagocytic activity in clearing the carbon particle, and birds from Group D and E had decreased phagocytic activity in clearing the carbon particle. The phagocytic activity of carbon particles after 3 and 15 minutes was highest in Group A, but the values were lowest, indicating delayed carbon clearance. The values were increased, but the phagocytic response decreased when the fipronil dose rate was increased. A similar result was examined after 15 min and 30 min, which are identical [48, 50]. The inflammatory response was decreased because the cells that reached the target site were immobilized. All the immune parameters observed during this trial have indicated that fipronil leads to immunotoxicity even at sub-lethal doses. It has been documented that fipronil induces immunotoxicity through free radical production in various tissues along with mitochondrial injury that leads to cell death [52, 53]. The immune system plays a crucial role in maintaining the health system of humans and animals. Such xenobiotics, including fipronil and other pesticides, not only lead to immunosuppression but pose serious threats, including genetic abnormalities, hormonal disruption, cancer, etc. [27, 54].

The deleterious effects of fipronil have been reported on various organs. The result of the study is discussed below, as the immune organs were part of it. The result revealed that the weight of the spleen was inversely proportional to the dose of the pesticide; as the dose of the pesticide increased, the weight of the spleen decreased. The lowest absolute weight of the spleen was recorded in group E, and it was significantly lower on the experiment's 15, 30^th^, and 45^th^ days. However, the relative weight of the spleen on the 15^th^ day of the experiment indicated that the control group had a non-significantly higher relative weight than all fipronil-treated groups, and the same trend was observed on the 30 and 45^th^ days of the experiment. The other two organs, the thymus and bursa of the Fabricius, showed the same trend as that of the spleen in this trial. The results were in accordance with those previously reported by Bano and Mohanty [23] who noted that fipronil toxicity results in a decrease in functional parenchyma of the immune organs, leading to lower immune cell production, so the size of the immune organs is regressed as compared to the normal.

Histopathology of these organs was performed during this trial, and significant findings were recorded. Group A (control) showed the normal structure of the spleen; no change was observed on days 15^th^, 30^th^, and 45^th^. White pulp, red pulp central arterioles, and capsule showed no changes. There is also a lymphoid follicle composed of B-lymphocytes. In high-dose treated group E, depletion of lymphocytes and infiltration of inflammatory cells were evident. The loss of lymphoid cells due to cell-mediated immune response suppression, a decrease of the immune cell type, and an alteration in average hormone values also proposed that immunosuppression existed due to functional fault in immunocompetent cells [22].

Group A (Control) showed the normal structure of the bursa of Fabricius; no change was observed on experimental days 15^th^, 30^th,^ and 45^th^. The lymphoid follicle showed the normal cortex (C), medulla (M), and cortico-medullary border. Group B showed mild congestion and disruption in the cortex (C), medulla (M), and cortico-medullary border, but severe congestion and disruption were observed in the C and D groups. In the last high-treated group, E showed severe congestion, infiltration of inflammatory cells, and follicular spacing were increased. Edema, lymphocytic depletion in the medulla and cortex, and mild interfollicular fibrosis in the bursa of Fabricius due to pesticide toxicity have already been reported [47, 55].

In the thymus, group A (Control) showed a normal structure, cell structure. No changes were observed on days 15^th^, 30^th,^ and 45^th^. Clearly, demarcated Hassall’s capsules were present. Group B showed mild changes and disruptions almost similar to those of the A group (Control), but severe congestion was observed in the C and D groups. Group E showed degenerative changes in the medullary region and depletion of lymphoid cells and was easily differentiated from control group A.

Lymphocyte cells are related to adaptive immune responses, as they produce immunoglobulins and recruit other cell types, thus modulating the immune response to specifically react to a given pathogen challenge [56, 57]. Diminishing numbers of lymphocytes, therefore, directly impact the adaptive responses of animals to diseases. As such, the lymphopenia detected in *O. carvalhoi* tadpoles exposed to chlorpyrifos reveals immunosuppression provoked by that xenobiotic, with consequent diminishing resistance to pathogens that could compromise their survival [57]. Similarly, most of the insecticides lead to leukopenia and lymphopenia, and sometimes, a decrease in the oxidative metabolic activity of phagocytes, which indicates immunosuppression [58-61]. Lymphopenia could be caused by insecticide destroying this series of cells resultantly loss of lymphocytes occurs [62,63]. Cytotoxic effects of pesticides/insecticides on T-lymphocytes lead to the depletion of these lymphoid cells, which ultimately results in immunosuppression [64-67].

**Conclusion**

It has been concluded that the fipronil toxicity at the sub-lethal level is also significant, leading to immunosuppression in non-target species when they are exposed to residues in the food chains. In poultry birds, these sub-lethal doses have immune-toxicological effects on humoral, cell-mediated, and non-specific immune responses. The toxic effects on the major immune organs in broilers in terms of decreased organ weight have also been recorded as playing a crucial role in immunosuppression.

**Author contribution**

Study concept and experimental design: STG, MKS, RH and AK.

Experimental execution and laboratory work: STG, MZT, BM and AK.

Data analysis and interpretation: STG, MZT, and AK.

Review and editing: STG, LA, RH and AK.

**Funding** No funds were provided by any funding agency. All the expenses were supported

by authors.

**Acknowledgement**

**Declarations**

**Consent to participate** It is declared that all the authors agreed to participate in editing this article.

**Consent for publication** It is the collective consent of all the authors to publish this data.

**Conflict of interest** The authors declare no competing interests.

**Data Availability Statement:** All data are in the manuscript and supporting information files.

**References**

1. Ahmad L, Gul ST, Saleemi MK, Hussain R, Rehman AU, Naqvi SNH, Du XX, Khan A. The effect of different repeated doses of cypermethrin on the behavioral and histological alterations in the brain of rabbits (*Oryctolagus cuniculi*). Int J Vet Sci. 2021; 10(4):347-54. <https://doi.org/10.47278/journal.ijvs/2021.092>
2. Zahoor MA, Gul ST, Khatoon A, Ashraf M, Zubair M, Imran M, Maqbool B, Atif FA. Teratogenic effects of thiamethoxam (a neonicotinoid) on development of chick embryo. Pak Vet J. 2022; 42(2):179-84.
3. Naseem S, Ghaffar A, Hussain R, Khan A. Inquisition of Toxic effects of Pyriproxyfen on physical, hemato-biochemical and histopathological parameters in Labeo rohita fish. Pak Vet J. 2022; 42(3):308-15.
4. Gunasekara AS, Truong T, Goh KS, Spurlock F, Tjeerdema RS. Environmental fate and toxicology of fipronil. J Pestic Sci. 2007; 32. <https://doi.org/10.1584/jpestics.R07-02>
5. Zhang Q, Wang X, Wang Q, Qi D, Huang Y, Chen R, Shan Y, Fang W. Toxicological effects of fipronil on laying hens and its residue elimination in eggs. Environ Sci Pollut Res. 2023; 30:19304-12. doi: 10.1007/s11356-022-23506-7. Epub 2022 Oct 13. PMID: 36227495.
6. Godinho AF, de Oliveira Souza AC, Carvalho CC, Horta DF, Fraia DD, Anselmo F, Chaguri JL, Faria CA. Memory impairment due to fipronil pesticide exposure occurs at the GABAA receptor level, in rats. Physiol Behav. 2016; 165:28–34. doi: 10.1016/j.physbeh.2016.06.035. Epub 2016 Jun 30. PMID: 27374426.
7. Lautz LS, Stoopen G, Ginting AJ, Hoogenboom RLAP, Punt A. Fipronil and fipronil sulfone in chicken: From in vitro experiments to in vivo PBK model predictions. Food Chem Toxicol. 2022; 165:113086. doi: 10.1016/j.fct.2022.113086. Epub 2022 Apr 29. PMID: 35500697.
8. Overmyer JP, Rouse DR, Avants JK, Garrison AW, Delorenzo ME, Chung KW, Key PB, Wilson WA, Black MC. Toxicity of fipronil and its enantiomers to marine and freshwater non-targets. J Environ Sci Heal - Part B Pestic Food Contam Agric Wastes. 2007. 42:471–80. <https://doi.org/10.1080/03601230701391823>
9. Qin F, Gao Y, Xu P, Guo B, Li J, Wang H. Enantioselective bio-accumulation and toxic effects of fipronil in the earthworm Eisenia foetida following soil exposure. Pest Manage Sci. 2015; 71:553–6. <https://doi.org/10.1002/ps.3841>
10. Singh NS, Sharma R, Singh SK, Singh DK. A comprehensive review of environmental fate and degradation of fipronil and its toxic metabolites. Environ Res. 2021; 199:111316. doi: 10.1016/j.envres.2021.111316. Epub 2021 May 11. PMID: 33989624.
11. Corrias F, Atzei A, Taddeo R, Arru N, Casula M, Salghi R, Russo M, Angioni A. Fipronil and fipronil sulfone distribution in chicken feathers and eggs after oral and dermal exposure. Foods. 2021; 10(12):3077. doi: 10.3390/foods10123077. PMID: 34945631; PMCID: PMC8702044.
12. Chen D, Li J, Zhao Y, Wu Y. Human exposure of fpronil insecticide and the associated health risk. J Agric Food Chem. 2022; 70:63–71. doi: 10.1021/acs.jafc.1c05694. Epub 2021 Dec 31. PMID: 34971309.
13. Bonmatin JM, Giorio C, Girolami V, Goulson D, Kreutzweiser DP, Krupke C, Liess M, Long E, Marzaro M, Mitchell EAD, Noome DA, Simon-Delso N, Tapparo A. Environmental fate and exposure; neonicotinoids and fipronil. Environ Sci Pollut Res. 2015; 22:35–67. doi: 10.1007/s11356-014-3332-7. Epub 2014 Aug 7. PMID: 25096486; PMCID: PMC4284396.
14. Wilson WA, Konwick BJ, Garrison AW, Avants JK, Black MC. Enantioselective chronic toxicity of fipronil to Ceriodaphnia dubia. Arch Environ Contam Toxicol. 2008; 54:36-43. doi: 10.1007/s00244-007-9003-7. PMID: 17687584.
15. Mohammed AT, Imam TS, Farag MR, Ghoneim MH. Assessment of the toxic impacts of acute exposure to fipronil insecticide on Japanese quails. Jpn J Vet Res. 2016; 64:S243–S249.
16. Kitulagodage M, Isanhart J, Buttemer WA, Hooper MJ, Astheimer LB. Fipronil toxicity in northern bobwhite quail, Colinus virginianus: reduced feeding behaviour and sulfone metabolite formation. Chemosphere. 2011; 83(4):524-30. doi: 10.1016/j.chemosphere.2010.12.057. Epub 2011 Jan 11. PMID: 21227481.
17. Bharatiya R, Bratzu J, Lobina C, Corda G, Cocco C, De Deurwaerdere P, Argiolas A, Melis MR, Sanna F. The pesticide fipronil injected into the substantia nigra of male rats decreases striatal dopamine content: A neurochemical, immunohistochemical and behavioral study. Behav Brain Res. 2020; 384:112562. doi: 10.1016/j.bbr.2020.112562. Epub 2020 Feb 15. PMID: 32070689.
18. Park H, Lee JY, Park S, Song G, Lim W. Developmental toxicity of fipronil in early development of zebrafish (Danio rerio) larvae: Disrupted vascular formation with angiogenic failure and inhibited neurogenesis. J Hazard Mater. 2020; 385:121531. doi: 10.1016/j.jhazmat.2019.121531. Epub 2019 Oct 30. PMID: 31732348.
19. Ghazanfar M, Shahid S, Qureshi IZ. Vitamin C attenuates biochemical and genotoxic damage in common carp (Cyprinus carpio) upon joint exposure to combined toxic doses of fipronil and buprofezin insecticides. Aquat Toxicol. 2018; 196:43-52. doi: 10.1016/j.aquatox.2017.12.015. Epub 2018 Jan 5. PMID: 29331520.
20. Xu H, Liu X, Jia Y, Dong F, Xu J, Wu X, Yang Y, Zheng Y. Fipronil-induced toxic effects in zebrafish (Danio rerio) larvae by using digital gene expression profiling. Sci Total Environ. 2018; 639:550-59.  doi: 10.1016/j.scitotenv.2018.05.159. Epub 2018 May 26. PMID: 29800848.
21. Montanha PF, Faria CA, Machado FD, Anselmo F, Silveira TL, Rocha NS, Godinho AF. Behavioral and laboratory abnormalities in rat offspring exposed to fipronil during the perinatal period. Int J Adv Vet Anim Res. 2018; 7:356-73.
22. Somwanshi AG, Karande VV, Jadhav ND, Gatne MM. Evaluation of immunotoxic potential of fipronil in wistar rats with special reference to cellular immune response. Int J Sci Environ Technol. 2018; 7:1700-710.
23. Bano F, Mohanty B. Thyroxine modulation of immune toxicity induced by mixture pesticides mancozeb and fipronil in mice. Life Sci. 2020; 240:117078. doi: 10.1016/j.lfs.2019.117078. Epub 2019 Nov 20.
24. Lovinskaya AV, Kolumbayeva SZ, Kolomiets OL, Abilev SK. [Genotoxic effects of pesticide fipronil in somatic and generative cells of mice]. Genetika. 2016; 52(5):561-8. Russian. PMID: 29368479.
25. Kadirovich TN, Bakhodirovna ZN. The influence of in utero and early postnatal exposure to pesticides on the process of cells apoptosis and proliferation in immune and endocrine organs of the offspring. Eur Sci Rev. 2017; 3-4:57-9.
26. El-Murr AE, Imam TS, Hakim Y, Ghonimi WA. Histopathological, immunological, hematological and biochemical effects of fipronil on Niletilapia (*Oreochromis niloticus*). J Vet Sci Technol. 2015; 6(5):2–9.
27. Akram R, Ghaffar A, Hussain R, Khan I, Santana VLDA, Mehmood K, Naz S, Iqbal R, Imran HM, Qamar MR, Zhu H. Hematological, serum biochemistry, histopathological and mutagenic impacts of triclosan on fish (bighead carp). Agrobiol Rec. 2022; 7:18-28. <https://doi.org/10.47278/journal.abr/2021.009>
28. Ardeshir RA, Zolgharnein H, Movahedinia A, Salamat N, Zabihi E. CYP1A gene expression as a basic factor for fipronil toxicity in Caspian kutum fish. Toxicol Reports. 2018; 5:113–24. doi: 10.1016/j.toxrep.2017.12.014. PMID: 29854583; PMCID: PMC5978015.
29. Chaguri JL, Godinho AF, Horta DF, Gonçalves-Rizzi VH, PossomatoVieira JS, Nascimento RA, Dias-Junior CA. Exposure to fpronil elevates systolic blood pressure and disturbs related biomarkers in plasma of rats. Environ Toxicol Pharmacol. 2016; 42:63–6. doi: 10.1016/j.etap.2015.12.020. Epub 2015 Dec 31. PMID: 26773360.
30. Timms L, Alexander DJ. Cell‐mediated immune response of chickens to Newcastle disease vaccines. Avian Path. 1977; 6(1):51-9. doi: 10.1080/03079457708418212. PMID: 18770312.
31. Shahzad A, Khan A, Khan MZ, Mahmood F, Gul ST, Saleemi MK. Immuno-pathologic effects of oral administration of chlorpyrifos in broiler chicks. J Immunotoxicol. 2015; 12:16–23. doi: 10.3109/1547691X.2013.866706. Epub 2013 Dec 16. PMID: 24328552.
32. Delhanty J, Solomon J. The nature of antibodies to goat erythrocytes in the developing chicken. Immunology. 1966; 11:103–13. PMID: 5920792; PMCID: PMC1423769.
33. Sattar A, Khan A, Hussain HI, He C, Hussain R, Zhiqiang S, Saleemi MK, Gul ST. Immunosuppressive effects of arsenic in broiler chicks exposed to Newcastle disease virus. J Immunotoxicol. 2016; 13:861-9. doi: 10.1080/1547691X.2016.1217105. Epub 2016 Aug 10. PMID: 27687888.
34. Zahid B, Saleem G, Aslam A, Imran MS, Younas M. Effect of immunostimulants on humoral response against infectious bursal disease in broilers. Pak Vet J. 2015; 35:227-30.
35. Sarker N, Tsudzuki M, Nishibori M, Yasue H, Yamamoto Y. Cell-medi-ated and humoral immunity and phagocytic ability in chicken lines diver-gently selected for serum immunoglobulin M and G levels. J Poult Sci. 2000; 79:1705-9. doi: 10.1093/ps/79.12.1705. PMID: 11194030.
36. Bancroft JD and Gamble M. Theory and practice of histological techniques, 6th ed. Churchil Livinsgstone, London, 2008; 80-186.
37. Mohammad LM, Kamil AM, Tawfeeq RK and Ahmed SJ, 2023. Ameliorating effects of herbal mixture for dexamethasone induced histological changes in mice. Int J Vet Sci. 2023; 12(1):126-31. <https://doi.org/10.47278/journal.ijvs/2022.170>
38. Abdelkhalek N, El‐Adl M, El‐Ashram A, Othman M, Gadallah H, Mohamed El-Diasty M, Dawood MAO,  Almeer R, Daim MA. Immunological and antioxidant role of curcumin in ameliorating fipronil toxicity in Nile tilapia (Oreochromis niloticus). Aquac Res. 2021; 52(6):1-11. <https://doi.org/10.1111/are.15131>
39. Khan I, Zaneb H, Masood S, Ashraf S, Rehman HF, Rehman HU, Ahmad S, Taj R, Salahuddin, Rahman SU. Supplemental selenium nanoparticles-loaded to chitosan improves meat quality, pectoral muscle histology, tibia bone morphometry and tissue mineral retention in broilers. Pak Vet J. 2022; 42(2):236-40.
40. Mahmood N, Rizvi F, Saleemi MK, Aslam MA. Seroprevalence and immunopathological studies of Salmonella pullorum in broiler birds in district Faisalabad Pakistan. Pak Vet J. 2022; 42(1):47-52.
41. Mashkoor J, Al-Saeed FA, Guangbin Z, Alsayeqh AF, Gul ST, Hussain R, Ahmad L, Mustafa R, Farooq U, Khan A. Oxidative stress and toxicity produced by arsenic and chromium in broiler chicks and application of vitamin E and bentonite as ameliorating agents. Front Vet Sci. 2023; 10:1128522. doi: 10.3389/fvets.2023.1128522. PMID: 36968473; PMCID: PMC10032408.
42. Adhikari D, Agarwal S. Effect of induced fipronil toxicity on humoral immune response in white leghorn cockerels. Int J Adv Res. 2019; 7:253–8. <http://dx.doi.org/10.21474/IJAR01/8321>
43. Gul ST, Khan RL, Saleemi MK, Ahmad M, Hussain R, Khan A. Amelioration of toxicopathological effects of thiamethoxam in broiler birds with vitamin E and selenium. Toxin Rev. 2022; 41(1):218-28. <https://doi.org/10.1080/15569543.2020.1864647>
44. Anwar U, Riaz M, Bilal Q, Yousaf M, Ashraf M, Aziz-ur-Rahman M. Impact of stored wheat-based feed on gut morphology, digesta viscosity and blood metabolites of broiler chickens. Pak Vet J. 2023; 43(1):179-83.
45. Vidau C, Brunet JL, Badiou A, Belzunces LP. Phenylpyrazole insecticides induce cytotoxicity by altering mechanisms involved in cellular energy supply in the human epithelial cell model Caco-2. Toxicol In Vitro. 2009; 23:589-97. doi: 10.1016/j.tiv.2009.01.017. Epub 2009 Feb 6. PMID: 19490841.
46. De Oliveira PR, Bechara GH, Denardi, SE, Oliveira RJ, Mathias MIC. Cytotoxicity of fipronil on mice liver cells. Microsc Res Tech. 2012; 75(1):28-35. doi: 10.1002/jemt.21018. Epub 2011 May 26. PMID: 21618652.
47. Gul ST, Khan A, Ahmad M, Ahmad H, Saleemi MK, Naseem MN, Bilal M (2019) Immuno-toxicological effects of different sub-lethal doses of thiamethoxam (TMX) in broiler birds. Toxin Rev. 2019; 38:200–205. <https://doi.org/10.1080/15569543.2018.1435554>
48. Gawade L, Dadarkar SS, Husain R, Gatne M. A detailed study of developmental immunotoxicity of imidacloprid in Wistar rats. Food Chem Toxicol. 2013; 51:61–70. doi: 10.1016/j.fct.2012.09.009. Epub 2012 Sep 18. PMID: 23000444.
49. Ghosh RC, Chauhan HVS, Jha GJ. Suppression of cell-mediated immunity by purified aflatoxin B1 in broiler chicks. Vet Immunol Immunopathol. 1991; 28:165–72. doi: 10.1016/0165-2427(91)90138-3. PMID: 1907777.
50. Gul ST, Khan A, Ahmad M, Anwar MF, Khatoon A, Saleemi MK, Akram MN. Effect of sub-lethal doses of thiamethoxam (a neonicotinoid) on hemato-biochemical parameters in broiler chicks. Toxin Rev. 2018; 37(2):144-8. <https://doi.org/10.1080/15569543.2017.1336731>
51. Morzadec C, Bouezzedine F, Macoch M, Fardel O, Vernhet L. Inorganic arsenic impairs proliferation and cytokine expression in humanprimary T-lymphocytes. Toxicology. 2012; 300:46-56. <https://doi.org/10.1016/j.tox.2012.05.025>
52. Vidau C, González-Polo RA, Niso-Santano M, Gómez-Sánchez R, Bravo-San Pedro JM, Pizarro-Estrella E, Blasco R, Brunet JL, Belzunces LP, Fuentes JM. Fipronil is a powerful uncoupler of oxidative phosphorylation that triggers apoptosis in human neuronal cell line SHSY5Y. Neurotoxicology. 2011; 32:935-43. <https://doi.org/10.1016/j.neuro.2011.04.006>
53. Aldayel TS, Abdel-Rahman HG, El-Hak HNG, Abdelrazek HM, Mohamed RM, El-Sayed RM. Assessment of modulatory activity of Uncaria tomentosa extract against fipronil immunotoxicity in male rats. Ecotoxicol Environ Saf. 2021; 224:112674. doi: 10.1016/j.ecoenv.2021.112674. Epub ahead of print. PMID: 34438272.
54. Swarnkar R, Anand S, Singh D, Choudhary A. Elucidation of immunomodulating potential of Morus alba against sub-acute exposure of Fipronil in rats. J Anim Res. 2019; 9(5):759-65.
55. Kammon AM, Brar RS, Banga HS, Sodhi S. Ameliorating effects of vitamin E and selenium on immunological alterations induced by imidacloprid chronic toxicity in chickens. J Environ Anal Toxicol. 2012; S4:007.
56. Davis AK, Maney DL, Maerz JC. The use of leukocyte profiles to measure stress in vertebrates: A review for ecologists. Funct Ecol. 2008; 22:760-72. <https://doi.org/10.1111/j.1365-2435.2008.01467.x>
57. da Silva MB, Fraga RE, Nishiyama PB, da Silva ISS, Costa NLB, de Oliveira LAA, Rocha MA, Juncá FA. Leukocyte Profiles in Odontophrynus carvalhoi (Amphibia: Odontophrynidae) Tadpoles Exposed to Organophosphate Chlorpyrifos Pesticides. Water Air Soil Pollut. 2020; 231:372. <https://doi.org/10.1007/s11270-020-04726-4>
58. Svoboda M, Luskova V, Drastichova J, Zlabek V. The effect of diazinon on haematological indices of common carp (Cyprinus carpio L.). Acta Vet Brno. 2001; 70:457–65.
59. Chandrasekara HU, Pathiratne A. Influence of low concentrations of Trichlorfon on haematological parameters and brain acetylcholinesterase activity in common carp, Cyprinus carpio L. Aquac Res. 2005; 36:144-9.
60. Narra MR, Rajender K, Reddy RR, Murty US, Begum G. Insecticides induced stress response and recuperation in fish: biomarkers in blood and tissues related to oxidative damage. Chemosphere. 2017; 168:350–57. doi: 10.1016/j.chemosphere.2016.10.066. Epub 2016 Oct 27. PMID: 27810534.
61. Bojarski1 B, Witeska M. Blood biomarkers of herbicide, insecticide, and fungicide toxicity to fish—a review. Environ Sci Pollut Res Int. 2020; 27(16):19236-50. doi: 10.1007/s11356-020-08248-8. Epub 2020 Apr 4. PMID: 32248419.
62. Fekry E, Awny MM, Makary S, El-Metwally AA. Protective effect of Pentoxifylline versus Nigella sativa against Cyclophosphamide induced splenic damage in adult male albino rats. Mansoura J Forensic Med Clinical Toxicol. 2023; 31(1):55-73.
63. Mondal SA, Ghosh RC, Mate MA, Ghosh CK. In-vivo sub-acute oral acetamiprid toxicity on hematological indices in Rattus norvegicus. Environ Ecol. 2009; 27:1767-9.
64. Krishnamoorthy P, Vairamuthu S, Balachandran C, Muralimanohar B. Pathology of lymphoid organs in chlorpyriphos and T-2 toxin fed broiler chicken. Int J Poult Sci. 2007; 6(1):71-6.
65. Singh ND, Sharma AK, Dwivedi P, Patil RD, Kumar M. Citrinin and endosulfan induced maternal toxicity in pregnant Wistar rats: pathomorphological study. J Appl Toxicol. 2007; 27(6):589-601. doi: 10.1002/jat.1242. PMID: 17429798.
66. Bernal-González KG, Covantes-Rosales CE, Camacho-Pérez MR, Mercado-Salgado U, Barajas-Carrillo VW, Girón-Pérez DA, Montoya-Hidalgo AC, Díaz-Resendiz KJG, Barcelos-García RG, Toledo-Ibarra GA, Girón-Pérez MI. Organophosphate-Pesticide-Mediated Immune Response Modulation in Invertebrates and Vertebrates. Int J Mol Sci. 2023; 24(6):5360. doi: 10.3390/ijms24065360. PMID: 36982434; PMCID: PMC10049729.
67. Brandt A, Hohnheiser B, Sgolastra F, Bosch J, Meixner MD, Büchler R. Immunosuppression response to the neonicotinoid insecticide thiacloprid in females and males of the red mason bee Osmia bicornis L. Sci Rep. 2020; 10(1):4670. doi: 10.1038/s41598-020-61445-w. PMID: 32170171; PMCID: PMC7070012.

**Table 1** Antibody titers of the broiler birds against Newcastle Disease virus treated with different doses of fipronil

| Groups | Experimental Days | | | |
| --- | --- | --- | --- | --- |
|  | Day 14^th^ | Day 21^st^ | Day 28^th^ | Day 35^th^ |
| A (control) | 170.66 ± 43.90^a^ | 213.33 ± 73.90^a^ | 106.66 ± 16.95^a^ | 85.33 ± 8.95^a^ |
| B | 106.66 ± 36.95^ab^ | 117.33 ± 21.15^ab^ | 26.66 ± 9.23^b^ | 53.33 ± 9.47^ab^ |
| C | 42.66 ± 18.47^b^ | 42.66 ± 18.47^ab^ | 13.33 ± 4.61^b^ | 5.33 ± 2.31^bc^ |
| D | 37.33 ± 24.44^b^ | 18.66 ± 12.22^b^ | 14.66 ± 15.14^b^ | 5.33 ± 2.30^bc^ |
| E | 21.33 ± 9.23^b^ | 10.66 ± 4.61^b^ | 5.33 ± 2.31^b^ | 3.33 ± 1.15^c^ |

Values (Mean ± SD) bearing different letters in a column differ

significantly (P≤0.05). Groups A, B, C, D, and E received fipronil @ 0 (control), 1.5, 2.5, 3.5, and 4.5 mg/kg body weight daily through crop tubing for 45 days.

**Table 2** Antibody titers against SRBCs of broiler birds treated with different doses of

Fipronil

| Parameter/Groups | Day 7^th^ (after primary injection) | Day 14^th^ (after primary injection + booster dose) | Day 21^st^ (after 7^th^ day of booster dose) | Day 28^th^ (after 14^th^ day of booster dose) |
| --- | --- | --- | --- | --- |
| Total Antibody Titers against SRBCs | | | | |
| A (control) | 7.66 ± 1.54^a^ | 7.33 ± 0.57^a^ | 8.33 ± 1.15^a^ | 5.66 ± 1.15^a^ |
| B | 6.33 ± 1.52^ab^ | 5.66 ± 0.58^ab^ | 5.66 ± 0.57^b^ | 4.33 ± 0.57^ab^ |
| C | 5.33 ± 1.55^ab^ | 4.33 ± 1.53^bc^ | 5.33 ± 0.58^b^ | 3.00 ± 0.58^bc^ |
| D | 4.66 ± 0.57^b^ | 3.66 ± 0.58^bc^ | 4.33 ± 0.57^b^ | 2.33 ± 0.57^c^ |
| E | 4.00 ± 0.58^b^ | 2.66 ± 0.57^c^ | 4.00 ± 0.58^b^ | 1.66 ± 0.59^c^ |
| IgG values against SRBCS | | | | |
| A (control) | 4.00 ± 1.00^a^ | 3.67 ± 1.52^a^ | 4.33 ± 1.51^a^ | 2.66 ± 0.57^a^ |
| B | 3.66 ± 1.15^ab^ | 3.33 ± 0.57^ab^ | 3.34 ± 0.58^ab^ | 1.67 ± 0.58^ab^ |
| C | 3.33 ± 2.08^ab^ | 2.66 ± 0.58^ab^ | 3.33 ± 0.57^ab^ | 1.66 ± 0.56^ab^ |
| D | 2.66 ± 0.57^ab^ | 2.33 ± 0.57^ab^ | 3.00 ± 1.00^ab^ | 1.00 ± 0.59^b^ |
| E | 2.33 ± 0.58^ab^ | 1.33 ± 0.58^b^ | 2.66 ± 0.58^ab^ | 1.01 ± 0.58^b^ |
| IgM values against SRBCS | | | | |
| A (control) | 3.66 ± 0.57^a^ | 3.66 ± 1.52^a^ | 4.00 ± 0.64^a^ | 3.00 ± 1.00^a^ |
| B | 2.66 ± 0.58^ab^ | 2.33 ± 0.57^ab^ | 2.33 ± 0.57^ab^ | 2.67 ± 0.57^a^ |
| C | 2.00 ± 1.00^ab^ | 1.66 ± 1.15^ab^ | 2.00 ± 0.58^ab^ | 1.33 ± 0.58^ab^ |
| D | 2.00 ± 0.57^ab^ | 1.33 ± 0.58^ab^ | 1.34 ± 0.57^ab^ | 1.33 ± 0.57^ab^ |
| E | 1.66 ± 0.58^b^ | 1.33 ± 0.57^ab^ | 1.33 ± 0.58^ab^ | 0.66 ± 0.58^b^ |

Values (Mean ± SD) bearing different letters in a column under specific parameters differ

significantly (P≤0.05). Groups A, B, C, D, and E received fipronil @ 0 (control), 1.5, 2.5, 3.5, and 4.5 mg/kg body weight daily through crop tubing for 45 days.

**Table 3** Immune organs' absolute and relative weight in birds different experimental days organs treated with different doses of fipronil

| Organs/ Groups | 15^th^ day | | 30^th^ day | | 45^th^ day | |
| --- | --- | --- | --- | --- | --- | --- |
|  | Absolute weight | Relative  weight | Absolute weight | Relative  weight | Absolute weight | Relative  weight |
| **Spleen** | | | | | | |
| A(Control) | 0.53 ± 0.07^a^ | 0.11 ± 0.02^a^ | 1.88 ± 0.05^a^ | 0.12 ± 0.03^a^ | 4.08 ± 0.06^a^ | 0.15 ± 0.04^a^ |
| B | 0.44 ± 0.14^ab^ | 0.10 ± 0.03^ab^ | 1.19 ± 0.22^b^ | 0.08 ± 0.02^ab^ | 3.32 ± 0.54^ab^ | 0.14 ± 0.03^ab^ |
| C | 0.31 ± 0.01^b^ | 0.07 ± 0.01^ab^ | 1.03 ± 0.06^b^ | 0.08 ± 0.01^b^ | 2.78 ± 0.47^bc^ | 0.12 ± 0.02^bc^ |
| D | 0.26 ± 0.08^b^ | 0.06 ± 0.02^ab^ | 1.04 ± 0.28^b^ | 0.07 ± 0.01^b^ | 2.23 ± 0.18^c^ | 0.11 ± 0.01^bc^ |
| E | 0.23 ± 0.06^b^ | 0.05 ± 0.01^ab^ | 0.86 ± 0.12^b^ | 0.06 ± 0.05^b^ | 1.97 ± 0.26^c^ | 0.10 ± 0.01^c^ |
| **Bursa of Fabricius** | | | | | | |
| A(Control) | 0.94 ± 0.04^a^ | 0.18 ± 0.02^a^ | 0.88 ± 0.06^a^ | 0.05 ± 0.03^a^ | 0.99 ± 0.05^a^ | 0.04 ± 0.01^a^ |
| B | 0.74 ± 0.06^b^ | 0.16 ± 0.04^ab^ | 0.65 ± 0.11^ab^ | 0.05 ± 0.01^ab^ | 0.80 ± 0.07^ab^ | 0.03 ± 0.01^ab^ |
| C | 0.66 ± 0.05^bc^ | 0.15 ± 0.05^ab^ | 0.61 ± 0.10^b^ | 0.04 ± 0.04^ab^ | 0.60 ± 0.05^bc^ | 0.03 ± 0.03^ab^ |
| D | 0.55 ± 0.02^cd^ | 0.13 ± 0.02^bc^ | 0.56 ± 0.07^bc^ | 0.03 ± 0.08^ab^ | 0.45 ± 0.03^bc^ | 0.02 ± 0.02^ab^ |
| E | 0.45 ± 0.09^d^ | 0.11 ± 0.02^c^ | 0.37 ± 0.06^c^ | 0.02 ± 0.03^b^ | 0.38 ± 0.02^c^ | 0.01 ± 0.01^b^ |
| **Thymus** | | | | | | |
| A(Control) | 1.52 ± 0.12^a^ | 0.30 ± 0.04^a^ | 5.14 ± 0.15^a^ | 0.32 ± 0.01^a^ | 11.87 ± 0.70^a^ | 0.45 ± 0.03^a^ |
| B | 1.29 ± 0.27^b^ | 0.29 ± 0.01^ab^ | 4.16 ± 0.39^ab^ | 0.30 ± 0.02^ab^ | 9.03 ± 0.33^b^ | 0.39 ± 0.01^ab^ |
| C | 1.12 ± 0.17^bc^ | 0.26 ± 0.02^ab^ | 3.43 ± 0.50^ab^ | 0.24 ± 0.03^ab^ | 7.85 ± 0.64^bc^ | 0.36 ± 0.02^bc^ |
| D | 1.02 ± 0.74^cd^ | 0.23 ± 0.06^ab^ | 3.45 ± 0.63^ab^ | 0.23 ± 0.01^ab^ | 7.14 ± 0.97^cd^ | 0.35 ± 0.04^bc^ |
| E | 0.72 ± 0.41^d^ | 0.17 ± 0.09^ab^ | 2.66 ± 0.48^b^ | 0.19 ± 0.02^ab^ | 5.58 ± 0.69^d^ | 0.29 ± 0.03^c^ |

Values (Mean ± SD) bearing different letters in a column under specific parameters differ

significantly (P≤0.05). Groups A, B, C, D, and E received fipronil @ 0 (control), 1.5, 2.5, 3.5, and 4.5 mg/kg body weight daily through crop tubing for 45 days.

Thickness in milimeter

| Experimental Hours | A (control) | B | C | D | E |
| --- | --- | --- | --- | --- | --- |
| 24 hours | 1.25 | 1.04 | 0.94 | 0.74 | 0.6 |
| 48hours | 0.66 | 0.69 | 0.64 | 0.38 | 0.35 |
| 72 hours | 0.52 | 0.41 | 0.38 | 0.34 | 0.19 |

**Fig. 1** Lymphoproliferative response against avian tuberculin in different treatment groups against fipronil toxicity. Groups A, B, C, D, and E received fipronil @ 0 (control), 1.5, 2.5, 3.5, and 4.5 mg/kg body weight daily through crop tubing for 45 days. The cell-mediated immune response was assessed through a delayed-type hypersensitivity response to avian tuberculin following the method already described [34] by Zahid et al. (2015).

| Experimental Time | A(control) | B | C | D | E |
| --- | --- | --- | --- | --- | --- |
| 3 minutes | 46.16 | 68.01 | 74.95 | 80.17 | 84.61 |
| 15 minutes | 36.85 | 49.75 | 69.11 | 73.17 | 81.57 |

**Fig. 2** Phagocytic activity in clearing carbon particles treated with fipronil. Groups A, B, C, D, and E received fipronil @ 0 (control), 1.5, 2.5, 3.5, and 4.5 mg/kg body weight daily through crop tubing for 45 days.

**Fig. 3.** a) Photomicrograph of the spleen (Group A) at 15^th^ day showing normal white and red pulp, b) Group E (Fipronil @ 4.5 mg/kg BW) at 15^th^ day showing disruption in white and red pulp, c) Group E at 30^th^ day showing severe congestion (arrowheads), and d) Group E at 45^th^ depletion of lymphocytes (arrows). H & E Staining. Magnification 200X.

**Fig. 4.** a) Photomicrograph of the bursa of Fabricius (Group A) at the 15^th^ day showing normal cortex (arrowhead), medulla (asterisk), and cortico-medullary border, b) Group E (Fipronil @ 4.5 mg/kg BW) at 15^th^-day showing moderate infiltration of inflammatory cells, c) at Group E at 30^th^-day showing vacuolar degeneration (arrowheads), and d) Group E at 45^th^ vacuolar degeneration (arrowheads) and infiltration of inflammatory cells. H & E Stain. Magnification 200X.
