# Supplementary material for: Fipronil in sub-lethal doses leads to immuno-toxicological effects in broiler birds
Source: PLoS One. 2025 Jan 28;20(1):e0315915. doi: 10.1371/journal.pone.0315915 (PMC11774380; doi:10.1371/journal.pone.0315915)
Supplement: Attachment — Submitted filename: Check List for Revision PONE-D-24-46735.docx [file pone.0315915.s005.docx]

**Check List of Manuscript # PONE-D-24-46735-Rev-I**

**We have corrected all the points raised by the three reviewers.**

**Changes in the manuscript are shown in blue colored font.**

| **Reviewer-1: Points Raised** | **How dealt** |
| --- | --- |
| My main concern is the appropriateness of using ANOVA with Tukey's post hoc test for a dose-response study. In dose-response experiments, where we’re evaluating graded responses to increasing doses of an additive or toxic compound like fipronil, contrasts such as linear or quadratic (or higher-order contrasts, if relevant) often provide a more meaningful analysis than p diff. We can quantify the strength and nature of the dose-response relationship in a more biologically relevant manner. ANOVA with Tukey’s post hoc test is more appropriate for comparing unrelated groups rather than ordered dose levels. Furthermore, the authors should clearly state the trial novelty or research gap to be filled with this study, many of the citations references previous trials with overlapping objectives with the current study. | We used ANOVA with Tukey's post hoc after consultation with a statistician. |
| **Specific Comments** | **Modification/justification** |
| LN 24: I think you mean plant agriculture here, as opposed to animal agriculture. | Agriculture indicates plants, crops etc. |
| LN 30: “maintained” at 37C | Proper elaboration has been done |
| Line # 72-74. Repeated sentences. | The repeated line has been deleted |
| Line # 103. Correct the word, “de-position” | Corrected |
| LN 126: Add brand identifiers/vendor for the fipronil used in this trial. | Brand name and company for the Fipronil has been added |
| LN 130: Add chicken breed | The commercial broiler chicken (Ross 308) used has been added |
| LN 139: What type of mineral oil. | It was corn oil. |
| LN 143: Curious there is no mortality recorded over a 45 day period for birds challenged multiple times. | No mortality was recorded. As the sub-lethal doses were used to check the toxicity, the birds remained alive but had potential toxicity effects on many organs and parameters. |
| Ln 154: How was blood collected? Please state the number of birds per replicate and how many were selected for sampling per phase | Every time, 6 birds were selected for necropsy and blood sample collections at each sampling phase. Blood without anticoagulant was collected from the wing vein in gel clot tubes for the serum samples, and then the serum was separated and stored until analysis. |
| Results and discussion: Rather than A,B—E, use better treatment descriptors here and in tables. It is easier for the readers to follow your study this way. Where necessary, combine common data into a single paragraph e.g. absolute/relative weights of organs can be combined into one or two succinct paragraphs. | As suggested, we have written fipronil dose with each group in the results and discussion. We have tried to combine the results wherever it was possible. |
| Data table: Add exact p values for each response criteria, not a blanket p value in the footnote. | Just to have a comprehensive data appearance, lettering has been added, and a cumulative P value has been mentioned to show the significance level among treatment groups. |
| **Reviewer 2** | |
| Introduction: This section is too long. It can be shortened. Repetitive sections should be edited. | The article's length is within the journal's prescribed limits, so there is no need to shorten this section. The authors have tried to provide relevant information regarding pesticides and their impacts on birds and the environment. |
| Line 39: aminobutyric acid (GABA)?? | Corrected as γ-aminobutyric acid |
| Line 53-55: double typing | Duplication deleted |
| Line 122: Please write clearly where it is first used. | Now written as Newcastle disease |
| Line 140-143: Use a combination of abbreviations and full forms here or in the title of this section. | Full form has been incorporated in paragraph along with abbreviation |
| Line 153: Where was it purchased? Origin? Trademark if any | Source has been incorporated |
| Line 171: bursa of Fabricius | Corrected as suggested |
| Line 172: Formula? | Now mentioned clearly |
| Line 238: Here, the difference between control and group e seems insignificant. | Yes, it is already mentioned that the difference is non-significant |
| Line 253: Since the values here are shown graphically, at least (Mean ± SE) should be provided for all groups. | Now incorporated |
| Line 337: Please write clearly where it is first used. | Now incorporated |
| Line 355: These results are mostly related to the humoral immune system. | As per suggestions from all the reviewers, this has been modified to make it clearer. |
| Line-408 | This has been revised based on the results of the current study. |
| **Reviewer 3** | |
| Line # 72-74. Repeated sentences. | Repetition has been deleted |
| Line # 103. Correct the word, “de-position” | Corrected |
| Line # As the authors are going to evaluate sub-lethal dosage, therefore mention the LD50 for broilers with reference, to experimental birds and treatments sub-heading. | LD50 mentioned with reference now. |
| Line # 138-139. “Groups B, C, D, and E received fipronil @ 1.5, 2.5, 3.5, and 4.5 mg/kg body weight daily and these doses were selected on the basis of previous studies reported.” Add the reference. | Reference Added. |
| Line # 154-155. What is meant by post-treatment, please re-write the sentence and make it clear. | Now corrected |
| Line # 182 …… chicks/group received ink (1 mL/kg) injection…. Please mention the type of ink. | Pelikan ink |
| Line # 185-186. How much amount of blood samples were mixed in 4mL of 1% sodium citrate? | one ml of blood samples was mixed with 4ml of 1% sodium citrate. |
| Line # 238. ……. response (4.00 ± 1.00, however, IgG in birds of Groups………. Close the parentheses properly. | Parentheses has been closed. |
| Line # 371-375. Re-write the sentence. | This has been rewritten. |
| Line # 377 and many others. Write SRBCs instead of SRBC. | Corrected at all places |
| Line # 393. As only twice the word DTH has been used in the whole MS, therefore use its full form. | Full form has been mentioned at both places |
| Line # 397-399. In these sentences, mention the treatment groups. | Treatment groups has been added. |
| Line # 409. The result revealed…….. Correct as “the results reviled ………”. | The word reviled is used in different context so does not fit here. |
| Line # 412 and 415. Add suffix th with numbers. | Has been added where it was missing. |
| Line # 438. Re-write the sentence. | It has been rewritten. |
| Line # 444-446. Improve the sentence. | It has been improved. |
| Line # Write the full name of O. carvalhoi | Now incorporated the full name |
